# Dublin hospital workers’ mental health during the peak of Ireland’s COVID-19 pandemic

**DOI:** 10.1007/s11845-022-03056-0

**Published:** 2022-06-22

**Authors:** Conan Brady, Caoimhe Fenton, Orlaith Loughran, Blánaid Hayes, Martina Hennessy, Agnes Higgins, Declan M. McLoughlin

**Affiliations:** 1 Dept. of Psychiatry and Trinity College Institute of Neuroscience, Trinity College Dublin, St Patrick’s University Hospital, Dublin 8, Ireland; 2 Beaumont Hospital, Dublin 9, Royal College of Surgeons, Dublin 2, Ireland; 3 WellcomeTrust/Health Research Board Clinical Research Facility, Trinity College Dublin, St James’s Hospital, Dublin 8, Ireland; 4 School of Nursing and Midwifery, Trinity College Dublin, Dublin, Ireland

**Keywords:** COVID-19, Hospital staff, Mental health, Moral injury, Post-traumatic stress

## Abstract

**Background:**

Hospital-based healthcare workers have experienced significant psychological stressors during the COVID-19 pandemic.

**Aim:**

To evaluate the mental health of hospital workers during the third wave of the COVID-19 pandemic in Dublin, Ireland.

**Methods:**

Cross-sectional anonymous online survey of hospital workers (*n* = 377; 181 doctors (48.0%), 166 nurses (44.0%), 30 radiographers (8.0%)), collecting demographic information, COVID-19 exposure history and mental health measures.

**Results:**

There were significant differences between profession groups in gender, experience, COVID-19 infection history, exposure to COVID-19 positive acquaintances, and work areas. Moderate-severe post-traumatic stress disorder (PTSD) symptoms were found in 45.1% (95% CI 40.1–50.1%) of all participants; significantly fewer doctors reported moderate-severe PTSD symptoms (26%; 95% CI 22–36%). A World Health Organisation-5 Wellbeing Index (WHO-5) score ≤ 32, indicating low mood, was reported by 52% (95% CI 47–57%) of participants; significantly fewer doctors reported low mood (46%; 95% CI 39–53%). One-week suicidal ideation and planning were reported respectively by 13% (95% CI 10–16%) and 5% (95% CI 3–7%) of participants with no between-group differences. Doctors reported significantly less moral injury than other groups. There were no significant between-group differences regarding coping styles. Work ability was insufficient in 39% (95% CI 34–44%) of staff; no between-group differences.

**Conclusions:**

Dublin hospital workers reported high levels of PTSD symptoms, mood disturbance, and moral injury during the COVID-19 pandemic. Concerning levels of suicidal ideation and planning existed in this cohort. Differences in degrees of post-traumatic stress, moral injury, and wellbeing were found between profession groups, which should be considered when planning any supports.

**Supplementary information:**

The online version contains supplementary material available at 10.1007/s11845-022-03056-0.

## Introduction

Hospital-based healthcare workers (HCWs) across the world have been severely tested during the COVID-19 pandemic. In addition to the typical stresses and restrictions experienced by society during the crisis, HCWs have had to contend with occupational hazards such as heightened rates of infection among healthcare staff, cancellation of elective procedures, potential redeployment, severe staffing shortages due to illness and quarantine, and essential resource shortages, e.g. personal protective equipment (PPE), ventilators, and intensive care beds [1–5]. All of this has occurred in the context of significant discontent in Irish healthcare staff even before the outbreak. Prepandemic, only 50.5% of Irish doctors reported positive subjective well-being and over a third (35%) reported psychological distress in a recent study [6]. Several cross-sectional studies have demonstrated high levels of stress and burnout in other Irish healthcare professionals, including nurses and radiographers [7–9]. It seems plausible that the pandemic could worsen preexisting low mood and depression. In addition, the common scenario of hospital staff struggling to work in facilities overwhelmed by COVID-19 is one that may provide fertile ground for the development of symptoms of post-traumatic stress disorder (PTSD). There have been two prior coronavirus outbreaks in the twenty-first century: the severe acute respiratory syndrome (SARS) outbreak in 2002–2004 and the Middle East respiratory syndrome (MERS) outbreak since 2012 [10]. Both have been shown to be associated with higher levels of post-traumatic stress symptoms in hospital workers [11, 12]. With respect to Irish studies during the pandemic, one cross-sectional study in the south-east of Ireland early in the pandemic found a prevalence of 41.3% of hospital staff reporting concerning levels of PTSD symptoms [13]. Another study focusing on Irish radiographers found that 40% reported symptoms of burnout [14].

Of increasing interest is a moral injury in HCWs, which is the distress experienced when an individual witness or engages in acts that contradict their moral and ethical beliefs [15]. This concept originates from studies in military populations, but the severe difficulties in providing optimal care during a pandemic have been theorised to provoke similar reactions in HCWs [16, 17]. Surveys of USA hospital workers have demonstrated high levels of moral injury over the course of the COVID-19 pandemic, as has a large study in the UK’s National Health Service (NHS) [16, 18].

The effects of the COVID-19 pandemic on hospital workers have been widely studied. A recent systematic review showed estimated pooled prevalence of 31.1% (95% CI 25.7–36.8%) for depression, 56.5% (95% CI 30.6–80.5%) for acute stress, and 20.2% (95% CI 9.9–33.0%) for post-traumatic stress [19].

Considering these international findings, we aimed to estimate the levels of post-traumatic stress symptoms and well-being in hospital staff (namely doctors, nurses, radiographers, and healthcare assistants (HCAs)) in Dublin, Ireland, during the COVID-19 pandemic. We also appraised self-rated suicidal ideation and planning, moral injury, coping styles, perceptions about the pandemic, and work ability. Finally, we explored if there were differences in these measures between different clinical professional groups.

## Methods

### Study design and setting

This was a cross-sectional, online, anonymous survey. Ethical approval was granted by the St James’s Hospital and Tallaght University Hospital Joint Research Ethics Committee and the Beaumont Hospital Ethics (Medical Research) Committee. Data were collected online using Qualtrics Core XM (Qualtrics, USA).

Recruitment targeted doctors, nurses, radiographers, and healthcare assistants working in three large Dublin teaching hospitals (St James’s Hospital, Tallaght University Hospital, and Beaumont Hospital). These are three of the largest hospitals in the Republic of Ireland, treating patients in diverse areas of Dublin, the city that has experienced the highest case numbers in the state [20]. The survey was open for a 6-week period from the 29th of January to the 16th of March 2021, coinciding with the third wave of Ireland’s COVID-19 outbreak. Per capita case numbers during the survey period were among the global highest in the pandemic at that time [21]. The survey also coincided with the rollout of the national vaccination programme, which initially targeted hospital workers and residents of long-term care [22]. Participating hospitals were asked to provide information on the number of staff working in each discipline so that a survey response rate could be determined.

Staff were recruited via internal email distribution, hospital intranet advertisements, poster advertisements, social media channels, snowballing, and direct liaison with department heads. Convenience sampling was used, with staff self-selecting for participation. Participation was voluntary, with participant information provided and consent obtained at the beginning of the survey. Information directing participants towards psychological support was provided beforehand and following the survey exit.

### Measures

We recorded basic demographic information along with profession, living arrangements, and preexisting physical and mental health conditions. Years of experience were recorded, and staff were assigned to a “junior/intermediate” group (which included all non-consultant hospital doctors and all other staff groups with less than 10 years of experience) and a “senior” group (which included all medical consultants and those in other roles with 10 years or more of experience). Questions were included to assess the extent of exposure to COVID-19, such as areas of work, self-quarantine experience, COVID-19 infection history, and history of contact with COVID-19-positive acquaintances.

The Impact of Event Scale-Revised (IES-R), a 22-item scale, was used to assess symptoms of post-traumatic stress disorder over the past week with subdomains corresponding to the three symptom clusters of PTSD (hyperarousal, intrusion, and avoidance); a score ≥ 26 indicated moderate-severe symptoms [23]. The World Health Organisation’s Well-Being Index (WHO-5), a five-item measure, was used to assess staff wellbeing [24]. Staff was asked to rate how they have been feeling over the past 2 weeks; scoring 21–32 indicates likely low mood and a score ≤ 20 indicates likely depression. Two Likert scale items from the Columbia Suicide Severity Rating Scale (C-SSRS) were used to assess suicidal ideation and planning over the previous seven days; responses were dichotomised based on the presence/absence of suicidal ideation or planning [25]. Staff perceptions of work ability were assessed using the Work Ability Score (WAS) derived from the Work Ability Index (WAI), an occupational health instrument for identifying those in need of support. Participants were asked to score their current ability to manage work demands compared to their lifetime best on a scale of 1–10 [26]. A score ≤ 5 corresponds with insufficient perceived work ability. The moral injury was measured using the Moral Injury Events Scale (MIES) adapted for healthcare staff during the COVID-19 pandemic [15]. This 9-item scale was originally developed to assess moral injury in combat veterans. Staff was asked if they agreed with statements relating to moral injury over the course of the COVID-19 outbreak. This measure has three subscales: “perceived transgressions by others” (where staff believed they had witnessed others act in a way that violated their moral beliefs), “perceived transgressions by self” (where staff felt they had violated their own moral code), and “betrayal” (assessing perceived betrayal by previously trusted leadership). Validated cut-off scores have yet to be developed for this instrument.

The Brief Coping Orientation to Problems Experienced (Brief-COPE) Scale was used to appraise the staff’s adaptive (“approach”; range 12–48) and maladaptive (“avoidance”; range 12–48) coping responses; staff were asked to identify which coping styles they had used over the course of the pandemic [27]. This scale also includes items for humour and religion.

A 15-item survey adapted from a study assessing HCW perceptions of the severe acute respiratory syndrome (SARS) outbreak was adapted for the COVID-19 pandemic [28]. This comprised three Likert scale items for each of the following groups of perceptions: health fear, social isolation, doubts about protective equipment, adequacy of training, and support and job stress. Items were rated 1 to 6; higher scores indicated higher levels of dissatisfaction with each domain. An additional Likert scale item assessing altruistic acceptance of risk was included; this was rated 1 to 6 with higher scores indicating higher degrees of altruism. Altruism has been reported to mediate the psychological impact of viral disease outbreaks on HCWs [29].

A free-text response box was provided at the end of the survey; analyses of these qualitative data will be separately reported.

### Statistical analysis

Data were analysed in Excel (Microsoft, USA) and SPSS 26 (IBM, USA). Using a 95% confidence interval with a 5% margin of error, our minimum sample size was determined to be 318 based on previous literature demonstrating that 71% of hospital staff scored ≥ 26 on the IES-R during the COVID-19 pandemic [30]. We examined the demographic characteristics of the sample divided into three groups: doctors, nurses, and radiographers. Healthcare assistants were not included due to low response rates. These groups were further categorised based on cut-off scores for the WHO-5, IES-R, and WAS and the presence/absence of suicidal ideation or planning. Chi-square tests were used to analyse categorical variables and one-way ANOVAs for means. Post hoc analyses were performed for significant between-group differences. The significance level was set at 0.05. We did not adjust for multiple testing, but regression analysis was performed using a generalised linear model to adjust for significant differences in demographic features (i.e. gender and level of seniority), exposure to COVID-19-infected acquaintances, and areas of work (emergency department work or non-COVID-19 treatment area). Data are reported as means (standard deviation) and proportions (percentages) as appropriate. Significant pairwise differences in post hoc analyses are reported as mean differences (MD) with standard errors (SE).

## Results

In total, there were 390 respondents. Due to the low HCA response rate (*n* = 13), they were excluded for analysis, leaving 377 participants, comprising 181 doctors (48.0%), 166 nurses (44.0%), and 30 radiographers (8.0%). The participating hospitals’ staff censuses differed substantially with respect to their methodology in categorising staff. This made calculating an accurate response rate impossible, but we attempted to extrapolate an approximate response rate based on the provided figures. This extrapolation estimated a response rate of 6%.

### Demographics

The demographic characteristics of survey participants are summarised in Table 1. Most staff were female (76.7%), lived with family (76.1%), were of white ethnicity (90.5%), and reported a senior level of experience (65.3%). The majority of staff reported no preexisting physical or mental illness. There were significant differences between doctors, nurses, and radiographers in gender (*p* < 0.01) and experience (*p* < 0.01). Doctors were more likely to be male (*z* = 7.8; see Supplementary Table S1). More nurses reported a senior level of experience (*z* = 5.4).

**Table 1:** Demographic characteristics of healthcare workers by role

	Total	Doctors	Nurses	Radiographers	Chi-square	
	*n* (%)	*n* (%)	*n* (%)	*n* (%)	χ^2^	*P-*value
Total	377 (100%)	181 (48.0%)	166 (44.0%)	30 (8.0%)		
Age (years)						
≤ 30	91 (24.1%)	43 (23.8%)	37 (22.3%)	11 (36.7%)		
31–50	200 (53.1%)	94 (51.9%)	88 (53.0%)	18 (60.0%)		
≥ 51	86 (22.8%)	44 (24.3%)	41 (24.7%)	1 (3.3%)	9.34	0.05^a^
Gender						
Male	84 (22.3%)	72 (39.8%)	10 (6.0%)	2 (6.7%)		
Female	289 (76.7%)	107 (59.1%)	154 (92.8%)	28 (93.3%)		
Non-binary	2 (0.5%)	2 (1.1%)	0 (0.0%)	0 (0.0%)		
Prefer not to say	2 (0.5%)	0 (0.0%)	2 (1.2%)	0 (0.0%)	69.21	< 0.01^a^
Living arrangements						
Alone	39 (10.3%)	15 (8.3%)	23 (13.9%)	1 (3.3%)		
With family	287 (76.1%)	140 (77.3%)	129 (77.7%)	18 (60.0%)		
With roommates	51 (13.5%)	26 (14.4%)	14 (8.4%)	11 (36.7%)	17.08	0.10^a^
Ethnicity						
Asian/Asian Irish	19 (5.0%)	7 (3.9%)	12 (7.2%)	0 (0.0%)		
Black/Black Irish	3 (0.8%)	2 (1.1%)	1 (0.6%)	0 (0.0%)		
Middle Eastern/ME Irish	3 (0.8%)	3 (1.7%)	0 (0.0%)	0 (0.0%)		
Mixed race	1 (0.3%)	0 (0.0%)	1 (0.6%)	0 (0.0%)		
Other	1 (0.3%)	0 (0.0%)	1 (0.6%)	0 (0.0%)		
SE Asian/SE Asian Irish	6 (1.6%)	2 (1.1%)	4 (2.4%)	0 (0.0%)		
White—Irish/British/other	341 (90.5%)	164 (90.6%)	147 (88.6%)	30 (100%)		
Prefer not to say	3 (0.8%)	3 (1.7%)	0 (0.0%)	0 (0.0%)	4.05	0.13^d^
Level of experience						
Junior/Intermediate	131 (34.7%)	85 (47.0%)	33 (19.9%)	13 (43.3%)		
Senior	246 (65.3%)	96 (53.0%)	133 (80.1%)	17 (56.7%)	29.07	< 0.01
Physical illness—Preexisting^b^						
Cancer	1 (0.3%)	1 (0.6%)	0 (0.0%)	0 (0.0%)		
Cardiovascular disease	24 (6.4%)	10 (5.5%)	14 (8.4%)	0 (0.0%)		
Immunosuppression	8 (2.1%)	4 (2.2%)	2 (1.2%)	2 (6.7%)		
Metabolic disease	28 (7.4%)	8 (4.4%)	17 (10.2%)	3 (10.0%)		
Respiratory disease	36 (9.5%)	15 (8.3%)	18 (10.8%)	3 (10.0%)		
Other	35 (9.3%)	18 (9.9%)	17 (10.2%)	0 (0.0%)		
None	265 (70.3%)	131 (72.4%)	111 (66.9%)	23 (76.7%)	1.90	0.39^c^
Mental illness—Preexisting^b^						
Anxiety disorder	25 (6.6%)	7 (4.2%)	17 (10.2%)	1 (3.3%)		
Mood disorder	25 (6.6%)	16 (9.6%)	8 (4.8%)	1 (3.3%)		
Other	5 (1.3%)	1 (0.6%)	3 (1.8%)	1 (3.3%)		
None	331 (87.8%)	161 (89.0%)	143 (86.1%)	27 (90.0%)	0.78	0.68^c^

*ME*, Middle Eastern; *SE*, Asian: Southeast Asian

^a^Fisher’s exact test

^b^respondents could pick multiple answers

^c^dichotomised for analysis (presence or absence of a preexisting condition)

^d^dichotomised for analysis (“White” and “Non-White”)

### Exposure to COVID-19

HCW exposure to COVID-19 is presented in Table 2. Significant differences were found in the number of staff working in the emergency department (*p* < 0.01) and non-COVID-19 designated areas (*p* < 0.01). Fewer nurses reported working in the emergency department (*z* =  −6.7; see Supplementary Table S2). Fewer radiographers reported working in designated COVID-19-free areas (*z* =  −5.0).

**Table 2:** Healthcare worker exposure to COVID-19 by role

	Total	Doctors	Nurses	Radiographers	Chi-square	
	*n* (%)	*n* (%)	*n* (%)	*n* (%)	χ^2^	*P*-value
Total	377 (100%)	181 (48.0%)	166 (44.0%)	30 (7.9%)		
Work area^b^						
Emergency department	78 (20.7%)	51 (28.2%)	8 (4.8%)	19 (63.3%)	64.91	< 0.01
COVID-19 treatment area	128 (34.0%)	64 (35.4%)	49 (29.5%)	15 (50.0%)	5.49	0.06
Non-Covid 19 treatment area	339 (89.9%)	172 (95.0%)	137 (82.5%)	30 (100.0%)	26.34	< 0.01
Previously self-quarantined	173 (45.9%)	84 (46.4%)	73 (44.0%)	16 (53.3%)	0.93	0.63
Previous COVID-19 infection	120 (31.8%)	51 (28.2%)	53 (31.9%)	16 (53.3%)	7.51	0.02
Symptom severity (*n* = 120)						
No symptoms	19 (15.8%)	6 (11.8%)	8 (15.1%)	5 (31.3%)		
Mild	51 (42.5%)	26 (51.0%)	20 (37.7%)	5 (31.3%)		
Moderate	47 (39.2%)	19 (37.3%)	22 (41.5%)	6 (37.5%)		
Severe	3 (2.5%)	0 (0.0%)	3 (5.7%)	0 (0.0%)	7.15	0.27^a^
Symptom duration (weeks; *n* = 120)						
≤ 4	86 (71.7%)	40 (78.4%)	35 (66.0%)	11 (68.8%)		
5–8	27 (22.5%)	9 (17.6%)	13 (24.5%)	5 (31.3%)		
≥ 9	7 (5.8%)	2 (3.9%)	5 (9.4%)	0 (0.0%)	3.56	0.45^a^
Fully recovered, *n* (%) (*n* = 120)	94 (78.3%)	42 (82.4%)	38 (71.7%)	14 (87.5%)	2.39	0.34^a^
Exposure to COVID-19 positive acquaintances						
Colleagues/acquaintances	341 (87.4%)	166 (91.7%)	146 (88.0%)	29 (96.7%)		
Close friends	215 (55.1%)	102 (56.4%)	94 (56.6%)	19 (63.3%)		
Housemates	22 (5.6%)	15 (8.3%)	6 (3.6%)	1 (3.3%)		
Immediate family	97 (24.9%)	44 (24.3%)	47 (28.3%)	6 (20.0%)		
No contact	11 (2.8%)	7 (3.9%)	4 (2.4%)	0 (0.0%)	0.92	0.56^a^
Acquaintances hospitalised, *n* (%) (*n* = 365^c^)	150 (41.1%)	68 (39.1%)	75 (46.6%)	7 (23.3%)	6.21	0.045
Acquaintances died, *n* (%) (*n* = 366)	44 (12.0%)	22 (12.6%)	20 (12.3%)	2 (6.7%)	0.66	0.76^a^

^a^Fisher’s exact test

^b^participants could select multiple answers

^c^one participant did not answer

^d^dichotomised to contact and noncontact for analysis

The proportion of staff reporting a history of self-quarantining was 45.9%, with no significant difference between clinical groups. Overall, 31.8% of staff reporting having contracted COVID-19; radiographers were significantly more likely to report a positive history (60.5%, *p* = 0.02; *z* = 4.0; see Supplementary Table S3). Of these, most reported full recovery from COVID-19 (78%) and there were no significant differences between groups in terms of COVID-19 symptom severity, duration, and recovery. With respect to exposure via contacts, most had been exposed to COVID-19 via colleagues or acquaintances (87.4%) with no significant differences between groups. Of the 366 respondents who reported contact with COVID-19 positive acquaintances, significantly more nurses reported having contact with those who had been hospitalised due to COVID-19 (*z* = 6.7). As this question was relevant to only a subset of all participants, this variable was not included for regression analysis.

### Mental health measures

Mental health outcomes are presented in Table 3. The proportion of all staff meeting the criterion for moderate-severe symptoms of PTSD was 45.1% (95% CI 40.1–50.1%). Significantly fewer doctors reported moderate-severe symptoms (29.3%; *p* < 0.01; *z* =  −5.9, see Supplementary Table S4). Radiographers were numerically more likely to meet this cut-off (73.3%), but this was not significantly higher than the number of nursing staff reporting moderate-severe symptoms (57.2%). There were also significant differences between the groups on the three subdomains of PTSD, with doctors reporting significantly lower scores on the avoidance (*p* < 0.01), hyperarousal (*p* < 0.01), and intrusion (*p* < 0.01) symptom domains than both nurses and radiographers (see Supplementary Table S5). These differences remained significant following regression analysis for significant demographic and infection exposure variables (adjusted *p* < 0.01).

**Table 3:** Healthcare worker mental health outcomes by role

	Total	Doctors	Nurses	Radiographers	Chi-square		
	*n* = 377	*n* = 181	*n* = 166	*n* = 30	χ^2^	*P*	*P* (adj.)^d^
IES-R 22, moderate/severe symptoms, % (95% CI)^a^	45 (40–50)	29 (22–36)	57 (49–65)	73 (57–89)	37.81	< 0.01	< 0.01
WHO-5^a^							
Poor wellbeing, % (95% CI)	52 (47–57)	46 (39–53)	55 (47–63)	70 (54–86)	7.15	0.03	0.07
Likely major depression, % (95% CI)	28 (23–33)	26 (20–32)	30 (23–37)	37 (20–54)	1.72	0.43	0.69
Suicidal ideation, % (95% CI)^b^	13 (10–16)	15 (10–20)	11 (6–16)	7 (0–16)	2.09	0.38^c^	0.64
Suicidal planning, % (95% CI)^b^	5 (3–7)	6 (3–9)	4 (1–7)	0 (0–0)	2.04	0.31^c^	0.46
WAS, Insufficient, % (95% CI)^a^	39 (34–44)	34 (27–41)	46 (38–54)	37 (20–54)	5.62	0.06	0.27
					One-way ANOVA		
					*F*	*P*	*P* (adj.)^d^
IES-R, mean (SD)							
Total	25.0 (16.1)	19.7 (15.3)	29.2 (15.3)	33.1 (14.9)	21.29	< 0.01	< 0.01
Avoidance	9.2 (6.6)	7.3 (6.5)	10.6 (6.3)	12.9 (5.4)	17.16	< 0.01	< 0.01
Hyperarousal	6.2 (4.6)	4.9 (4.2)	7.3 (4.7)	7.8 (4.3)	14.47	< 0.01	< 0.01
Intrusion	9.6 (6.5)	7.6 (6.2)	11.4 (6.2)	12.4 (6.2)	19.73	< 0.01	< 0.01
MIES, mean (SD)							
Total	22.3 (9.8)	20.4 (9.6)	23.8 (9.8)	25.7 (9.5)	7.46	< 0.01	< 0.01
Transgression—others	6 (3.1)	5.6 (3.0)	6.4 (3.2)	6.7 (2.9)	3.94	0.02	< 0.01
Transgression—self	7.9 (4.5)	7.3 (4.3)	8.4 (4.7)	8.1 (3.7)	2.63	0.07	< 0.01
Betrayal	8.4 (4.3)	7.5 (4.0)	9 (4.3)	10.9 (4.8)	11.44	< 0.01	< 0.01
Brief-COPE, mean (SD)							
Avoidant	21.8 (5.3)	21.2 (5.4)	22.4 (5.1)	22.4 (5.7)	2.31	0.10	< 0.01
Approach	29.4 (6.6)	29.2 (6.4)	29.5 (6.7)	30.2 (6.5)	0.38	0.68	0.64
Religion	3.3 (1.7)	3.1 (1.5)	3.6 (1.9)	3.1 (1.2)	4.88	< 0.01	0.03
Humour	4.3 (1.9)	4.5 (2.0)	4.1 (1.9)	4.1 (1.8)	1.89	0.15	0.52
COVID-19 perceptions, mean (SD)							
Doubts about protection	1.7 (0.7)	1.7 (0.7)	1.6 (0.8)	1.4 (0.5)	2.64	0.07	0.18
Doubts about systems/processes	2.5 (1.0)	2.6 (0.9)	2.4 (1.0)	2.8 (1.0)	3.97	0.02	0.06
Health fear	4.8 (1.0)	4.5 (1.1)	4.9 (1.0)	5.3 (0.7)	12.85	< 0.01	< 0.01
Job stress	4.4 (1.1)	4.1 (1.1)	4.6 (1.0)	4.8 (0.9)	13.82	< 0.01	< 0.01
Social isolation and avoidance	3.4 (1.1)	3.1 (1.0)	3.6 (1.2)	3.4 (1.1)	12.69	< 0.01	< 0.01
Altruism perception, mean (SD)	4.9 (1.1)	4.9 (1.0)	4.8 (1.2)	5.1 (0.7)	1.42	0.24	0.75

*SD*, standard deviation; *95% CI*, 95% confidence interval; *WHO-5*, World Health Organisation-Five Wellbeing Index: maximum of 100; score of 33 or more indicates normal wellbeing over the past 2 weeks; 20 or less indicates likely depression over the past 2 weeks. *IES-R*, impact of events scale revised (22 items); cut-off of 26 or more indicates moderate-to-severe symptoms of post-traumatic stress over the past 7 days. Work ability score: maximum of 10; cut-off of 5 or less indicates insufficient perceived work ability. *MIES*, Moral Injury Events Scale. Higher scores denote higher intensity of moral injury over the course of the COVID-19 outbreak. Brief-COPE: abbreviated version of the COPE (Coping Orientation to Problems Experienced) Inventory. Scores range from 12 to 48 for avoidant/approach scales. Higher scores indicate greater reliance on this coping style over the course of the pandemic. Perceptions of health fear, social isolation and avoidance, job stress, dissatisfaction with system/processes, doubts about protection, and altruism. Higher scores indicate increased identification with each domain over the course of the COVID-19 outbreak

^a^Item dichotomised for analysis using cut-off score

^b^Items are dichotomised for analysis (any suicidal ideation/planning and none)

^c^Fisher’s exact test

^d^Following regression analysis adjusting for gender, seniority, history of COVID-19 infection, and areas of work

The whole cohort WHO-5 mean was 38.0 (21.6). Of all staff, 52% (95% CI 47–57%) met the criterion for low mood on the WHO-5, with 28% (95% CI 23–33%) reporting scores consistent with likely depression. A significant difference was present between professions reporting low mood (*p* = 0.03), with doctors being less likely to meet this criterion (*z* = –2.2) and radiographers being more likely (*z* = 2.1); these differences did not survive regression analysis, however (*p* = 0.07). There was no significant difference between groups for those below the “likely depression” cut-off. Suicidal ideation was reported by 13% (95% CI 10–16%) of all staff, and 5% (95% CI 3–7%) of staff reported at least some plans to end their life, with no significant differences between groups on either measure.

The whole cohort WAS mean was 5.9 (2.1). Insufficient work ability was reported by 39% (95% CI 34–44%) of staff with no differences between groups.

The mean MIES score was 22.3 (9.8). There were significant differences between groups on the total score (*p* < 0.01) and all three MIES subscales; these differences survived regression analysis (*p* < 0.01). Doctors reported significantly lower total MIES scores than both nurses (MD −3.5, SE 1.0) and radiographers (MD −5.3, SE 1.9; see Supplementary Table S6). Doctors also reported a significantly lower score on the “perceived transgressions by others” subscale than nurses (MD −0.8, SE 0.3) and a significantly lower score on the “Perceived betrayal” subscale than both nurses (MD −1.5, SE 0.45) and radiographers (MD −3.4, SE 0.8). Post hoc analysis of the “Transgressions by self” subscale did not reveal significant differences between groups in pairwise comparisons.

There were no significant differences between groups in their use of avoidant (maladaptive) and approach (adaptive) coping strategies prior to regression analysis; however, a significant difference was noted between groups on the avoidant scale following regression analysis (*p* < 0.01), which arose due to significant differences between gender groups (*p* < 0.01) and level of experience (*p* < 0.01; see Supplementary Table S7). Males were less likely to use avoidant coping strategies than females; junior staff was more likely to use avoidant coping strategies than senior staff. There were no differences between professions in their use of humour as a coping mechanism. Nurses were significantly more likely to use religion as a coping strategy than doctors (MD 0.54, SE 0.2, *p* < 0.01, adjusted *p* = 0.03; see Supplementary Table S8).

Staff tended to agree with statements regarding their fear of contracting COVID-19 and job stress. To a lesser extent, they also agreed with statements indicating a degree of concern about social stigma and social isolation in relation to work. They tended not to have concerns about personal protective equipment (PPE) or the system and processes established in their workplace. Significant differences were noted with respect to levels of health fear (*p* < 0.01), job stress (*p* < 0.01), and social isolation and avoidance (*p* < 0.01), all of which survived regression analysis. Doctors reported significantly less concern than both nurses and radiographers in all three domains (see Supplementary Table S9). Radiographers reported significantly more concern regarding health fear than nurses (MD 0.4, SE 0.1). Staff broadly agreed with an altruistic statement about accepting risks involved in caring for patients with COVID-19, with no significant differences between groups.

## Discussion

This study adds to a growing body of literature demonstrating the significant burden of mental health issues experienced by healthcare professionals globally during the COVID-19 pandemic. The strengths of this study include the use of validated assessment tools used, and the timing of the survey, which occurrs at the peak of Ireland’s pandemic to date. COVID-19 hospitalisations peaked in Ireland on January 18th, 2021 at 2020, and the number of ICU patients peaked on January 23rd at 221 [31, 32].

Our estimate for the 1-week prevalence of moderate-severe symptoms of PTSD in Irish hospital workers (45%, 95% CI 40–50%) during the COVID-19 pandemic was higher than the current best estimate for psychotraumatic disorders internationally (31.1%, 95% CI 25.7–36.8%) and the prevalence of probable PTSD in NHS workers in the UK (30.2%, 95% CI 28.1–32.5) [18, 19]. This is an important finding, potentially indicating that Irish hospital workers were at least as distressed—if not more so—than their global peers. However, these international studies are marked by heterogeneity in terms of measures and cut-offs used, making direct comparisons difficult. In addition, as our survey occurred at the peak of Ireland’s pandemic, our findings may reflect acute stress reactions to the events at that time rather than prolonged effects from the initial waves of the pandemic. Rigorous longitudinal studies are required to determine if these issues persist. Of note, professions differ significantly, with radiographers and nurses reporting worse scores than doctors. These factors survived regression analysis, indicating that these issues are role-specific rather than due to other factors.

The prevalence of likely depression in this Irish cohort (28%, 95% CI 28–33%) is similar to the estimated pooled prevalence of depression in a recent meta-analysis of global studies (31.1%, 95% CI 25.7–36.8%) [19]. There are no data on WHO-5 wellbeing scores in Irish nursing staff and radiographers prepandemic. However, the prevalences of low mood (46.0%, 95% CI 38.7–53.3%) and likely depression (26.0%, 95% CI 22.4–35.6%) for doctors during the pandemic were not significantly different from that reported prepandemic (i.e. low mood: 49.5%, 95% CI 47.2–41.8%; likely depression: 22.2%, 95% CI 20.3–24.1%) [6]. It is unclear whether this reflects the reality that doctors’ well-being has not changed due to the pandemic or if this is a result of our methodology. One explanation could be that there is a ceiling effect of the WHO-5 wellbeing index in this population. However, given that the mean WHO-5 score in this study is lower than that seen in other studies in various populations, this seems unlikely [24]. Another explanation could be a selection bias favouring doctors who have low moods but, again, this seems unlikely given the similar findings in a prepandemic study [6]. This interesting finding warrants further longitudinal studies to clarify if these issues persist in both doctors and other healthcare professionals.

The 1-week prevalences of suicidal ideation and planning concerning at 13 and 5%, respectively. International estimates of suicidal ideation and planning (as a combined prevalence) in hospital workers vary from 5.4 to 12%; however, these are 2-week prevalences [33–37]. Only one study to date has reported suicidal planning as a separate item in hospital workers; this Spanish study found the 30-day prevalence to be 2.7% [38]. These apparently high levels of suicidal thinking in hospital staff warrant further investigation.

Unfortunately, there are no prepandemic data available on work ability for Irish radiographers or nurses. The prevalence of doctors reporting insufficient work ability (39%, 95% CI 34–44%) was significantly higher than the prepandemic prevalence (29%, 95% CI 27.1–31.3%), indicating that doctors’ occupational functioning may have worsened [39]. However, it cannot be said for certain that this is due to the pandemic or to other factors.

Irish hospital workers report comparatively high levels of moral injury. The mean score of 22.3 (9.8) is significantly higher than that of US hospital staff at the beginning of the pandemic (MD 7.8, SE 1.1, *p* < 0.0001), and also significantly higher than that of NHS staff (MD 6.8, SE 0.149, *p* < 0.0001) [16, 18]. Again, doctors reported lower degrees of moral injury than other professions both before and after regression analysis. The largest mean differences were on the “betrayal” subscale; radiographers reported the highest numerical mean on this measure, indicating that they felt significantly higher levels of betrayal by previously trusted authority figures.

Regarding perceptions of the experience of the COVID-19 outbreak, views were similar to those reported by hospital workers during the SARS outbreak [28], with high levels of altruism reported. While professions report similar coping styles, this does not seem to have protected nurses and radiographers from higher levels of post-traumatic stress and moral injury.

### Limitations

There are several limitations to this study. Firstly, as this is a cross-sectional study, it is not possible to say that the issues identified across hospital workers are caused by the pandemic or if they simply represent a continuation of longstanding problems. Secondly, individual hospital data censuses of staff and working hours differ substantially in their breakdowns of staff by profession, leaving us unable to perform an accurate analysis of whether our data are representative of all staff. Thirdly, any cross-sectional study is prone to selection bias, and it is possible that participants responded based on the level of their psychological distress. Our extrapolated response is low at 6%, meaning that our findings are prone to selection bias. Unfortunately, this is a common limitation in studies examining HCW mental health; one recent large study in the UK made substantial efforts to avoid this issue and obtained a response rate of 12% [18]. However, the similarly high levels of distress seen in this and other studies would suggest that there is a commonality to the experience of hospital workers during the COVID-19 pandemic.

## Conclusions

In this survey performed during the peak of Ireland’s COVID-19 pandemic, the prevalence of moderate-severe post-traumatic stress and the degree of moral injury are at least as high as those of hospital workers globally. Significant differences exist between professions in terms of mental health outcomes, highlighting the need for tailored psychological supports to be devised for staff. Robust longitudinal studies are required to assess if these issues persist in easing the pandemic and related social restrictions.

## Supplementary information

Below is the link to the electronic supplementary material.

Supplementary file1 (DOCX 32 KB)
