# Supplementary material for: Dublin hospital workers’ mental health during the peak of Ireland’s COVID-19 pandemic
Source: Ir J Med Sci. 2022 Jun 22;192(3):1293–302. doi: 10.1007/s11845-022-03056-0 (PMC9217120; doi:10.1007/s11845-022-03056-0)
Supplement: Supplementary file 1 — Supplementary file1 (DOCX 32 KB) [file 11845_2022_3056_MOESM1_ESM.docx]

**Supplementary information**

# Supplementary Data Table of Contents

- S1: Post hoc analyses of demographic differences between groups, by role
- S2: Post hoc analyses of Covid-19 exposure differences between groups, by role: (i) Work areas; (ii) Personal Covid-19 exposure
- S3: Post hoc analyses of Covid-19 exposure history, by role
- S4: Post hoc analyses of significant differences in staff meeting IES-R and WHO-5 criteria, by role
- S5: Post hoc analyses of Impact of Events Scale-Revised (IES-R) subscale means; pairwise comparisons by role
- S6: Post hoc analyses of moral injury events scale (MIES) and subscale means; pairwise comparisons by role
- S7: Brief-COPE avoidant (maladaptive) subscale regression analysis for significant demographic and Covid-19 exposure variables
- S8: Post-hoc analysis of Brief-COPE religion subscale, pairwise comparisons by role
- S9: Post-hoc analyses of significant differences in perceptions scales; pairwise comparisons by roles

Supplementary Table S1: Post hoc analyses of demographic differences between groups, by role

|  | | | Doctors | Nurses | Radiographers |
| --- | --- | --- | --- | --- | --- |
| Gender | Male | Count | 72^a^ | 10^b^ | 2^b^ |
|  |  | Z-score | 7.8 | -6.7 | -2.1 |
|  | Female | Count | 107^a^ | 154^b^ | 28^b^ |
|  |  | Z-score | -7.7 | 6.6 | 2.3 |
|  | Non-binary | Count | 2^a^ | 0^a^ | 0^a^ |
|  |  | Z-score | 1.5 | -1.3 | -.4 |
|  | Prefer not to say | Count | 0^a^ | 2^a^ | 0^a^ |
|  |  | Z-score | -1.4 | 1.6 | -.4 |
| Level of Experience | Junior/Intermediate | Count | 85^a^ | 33^b^ | 13^a^ |
|  |  | Z-score | 4.8 | -5.4 | 1.0 |
|  | Senior | Count | 96^a^ | 133^b^ | 17^a^ |
|  |  | Z-score | -4.8 | 5.4 | -1.0 |

Each superscript letter denotes a subset of categories whose column proportions do not differ significantly from each other at the .05 level. Bonferroni correction applied.

**Supplementary Table S2: Post hoc analyses of work areas, by role**

|  | | Doctors | Nurses | Radiographers |
| --- | --- | --- | --- | --- |
| Percentage reporting working in emergency department: | Count | 51^a^ | 8^b^ | 19^c^ |
|  | Z-score | 3.4 | -6.7 | 6.0 |
| Percentage reporting working in non-Covid-19 designated areas: | Count | 162^a^ | 142^a^ | 16^b^ |
|  | Z-score | 2.4 | .3 | -5.0 |

Each superscript letter denotes a subset of categories whose column proportions do not differ significantly from each other at the .05 level. Bonferroni correction applied.

# Supplementary Table S3: Post hoc analyses of Covid-19 exposure history, by role

|  | | | Doctors | Nurses | Radiographers |
| --- | --- | --- | --- | --- | --- |
| History of Covid-19 infection in staff member | Yes | Count | 51^a^ | 53^a, b^ | 16^b^ |
|  |  | Z-score | -1.5 | .0 | 2.6 |
|  | No | Count | 130^a^ | 113^a, b^ | 14^b^ |
|  |  | Z-score | 1.5 | .0 | -2.6 |
| Contact with acquaintance who has been hospitalised due to Covid-19 (Of staff members reporting exposure to Covid-19 acquaintances, n=365) | Yes | Count | 68^a^ | 75^a^ | 7^a^ |
|  |  | Z-score | -.7 | 1.9 | -2.1 |
|  | No | Count | 106^a^ | 86^a^ | 23^a^ |
|  |  | Z-score | .7 | -1.9 | 2.1 |

Each superscript letter denotes a subset of categories whose column proportions do not differ significantly from each other at the .05 level.

**Supplementary Table S4: Post hoc analyses of significant differences in staff meeting IES-R and WHO-5 criteria, by role**

|  | | | Doctors | Nurses | Radiographers |
| --- | --- | --- | --- | --- | --- |
| IES-R | No/Mild Symptoms | Count | 128^a^ | 71^b^ | 8^b^ |
|  |  | Z-score | 5.9 | -4.2 | -3.2 |
|  | Moderate /Severe Symptoms | Count | 53^a^ | 95^b^ | 22^b^ |
|  |  | Z-score | -5.9 | 4.2 | 3.2 |
| WHO-5 | Poor wellbeing | Count | 83^a^ | 91^a, b^ | 21^b^ |
|  |  | Z-score | -2.2 | 1.1 | 2.1 |
|  | Normal wellbeing | Count | 98^a^ | 75^a, b^ | 9^b^ |
|  |  | Z-score | 2.2 | -1.1 | -2.1 |

Each superscript letter denotes a subset of categories whose column proportions do not differ significantly from each other at the .05 level.

Supplementary Table S5: Post hoc analyses of Impact of Events Scale-Revised (IES-R) subscale means; pairwise comparisons by role

|  |  |  | Mean Difference | Std. Error | *p* | 95% Confidence Interval | |
| --- | --- | --- | --- | --- | --- | --- | --- |
|  |  |  |  |  |  | Lower Bound | Upper Bound |
| Avoidance Subscale | Doctor | Nurse | -3.29^*^ | 0.68 | 0.00 | -4.89 | -1.69 |
|  |  | Radiographers | -5.56^*^ | 1.25 | 0.00 | -8.49 | -2.62 |
|  | Nurse | Doctor | 3.29^*^ | 0.68 | 0.00 | 1.69 | 4.89 |
|  |  | Radiographers | -2.27 | 1.26 | 0.17 | -5.22 | 0.68 |
|  | Radiographers | Doctor | 5.56^*^ | 1.25 | 0.00 | 2.62 | 8.49 |
|  |  | Nurse | 2.27 | 1.26 | 0.17 | -0.68 | 5.22 |
| Hyperarousal Subscale | Doctor | Nurse | -2.40^*^ | 0.48 | 0.00 | -3.53 | -1.27 |
|  |  | Radiographers | -2.89^*^ | 0.88 | 0.00 | -4.95 | -0.82 |
|  | Nurse | Doctor | 2.40^*^ | 0.48 | 0.00 | 1.27 | 3.53 |
|  |  | Radiographers | -0.49 | 0.88 | 0.84 | -2.57 | 1.59 |
|  | Radiographers | Doctor | 2.89^*^ | 0.88 | 0.00 | 0.82 | 4.95 |
|  |  | Nurse | 0.49 | 0.88 | 0.84 | -1.59 | 2.57 |
| Intrusion Subscale | Doctor | Nurse | -3.80^*^ | 0.67 | 0.00 | -5.38 | -2.24 |
|  |  | Radiographers | -4.89^*^ | 1.22 | 0.00 | -7.75 | -2.01 |
|  | Nurse | Doctor | 3.81^*^ | 0.67 | 0.00 | 2.24 | 5.38 |
|  |  | Radiographers | -1.07 | 1.23 | 0.66 | -3.96 | 1.82 |
|  | Radiographers | Doctor | 4.89^*^ | 1.22 | 0.00 | 2.01 | 7.75 |
|  |  | Nurse | 1.07 | 1.23 | 0.66 | -1.82 | 3.96 |

*The mean difference is significant at the 0.05 level.

Supplementary Table S6: Post hoc analyses of moral injury events scale (MIES) and subscale means; pairwise comparisons by role

|  |  |  | Mean Difference |  |  | 95% Confidence Interval | |
| --- | --- | --- | --- | --- | --- | --- | --- |
|  |  |  |  | Std. Error | *p* | Lower Bound | Upper Bound |
| MIES Total Score | Doctors | Nurses | -3.45^*^ | 1.04 | 0.00 | -5.91 | -1.00 |
|  |  | Radiographers | -5.32^*^ | 1.91 | 0.02 | -9.83 | -0.82 |
|  | Nurses | Doctors | 3.46^*^ | 1.04 | 0.00 | 1.00 | 5.91 |
|  |  | Radiographers | -1.87 | 1.93 | 0.60 | -6.40 | 2.66 |
|  | Radiographers | Doctors | 5.32^*^ | 1.91 | 0.02 | 0.82 | 9.83 |
|  |  | Nurses | 1.87 | 1.93 | 0.60 | -2.66 | 6.40 |
| Transgressions by others | Doctors | Nurses | -0.84^*^ | 0.33 | 0.03 | -1.62 | -0.06 |
|  |  | Radiographers | -1.10 | 0.61 | 0.17 | -2.53 | 0.32 |
|  | Nurses | Doctors | 0.84^*^ | 0.33 | 0.03 | 0.06 | 1.62 |
|  |  | Radiographers | -0.26 | 0.61 | 0.90 | -1.70 | 1.17 |
|  | Radiographers | Doctors | 1.10 | 0.61 | 0.17 | -0.32 | 2.53 |
|  |  | Nurses | 0.26 | 0.61 | 0.90 | -1.17 | 1.70 |
| Transgressions by self | Doctors | Nurses | -1.09 | 0.48 | 0.06 | -2.21 | 0.04 |
|  |  | Radiographers | -0.79 | 0.88 | 0.64 | -2.85 | 1.27 |
|  | Nurses | Doctors | 1.09 | 0.48 | 0.06 | -0.04 | 2.21 |
|  |  | Radiographers | 0.29 | 0.88 | 0.94 | -1.78 | 2.37 |
|  | Radiographers | Doctors | 0.79 | 0.88 | 0.64 | -1.27 | 2.85 |
|  |  | Nurses | -0.29 | 0.88 | 0.94 | -2.37 | 1.78 |
| Betrayal | Doctors | Nurses | -1.53^*^ | 0.45 | 0.00 | -2.59 | -0.47 |
|  |  | Radiographers | -3.43^*^ | 0.83 | 0.00 | -5.38 | -1.48 |
|  | Nurses | Doctors | 1.53^*^ | 0.45 | 0.00 | 0.47 | 2.59 |
|  |  | Radiographers | -1.90 | 0.83 | 0.06 | -3.86 | 0.06 |
|  | Radiographers | Doctors | 3.43^*^ | 0.83 | 0.00 | 1.48 | 5.38 |
|  |  | Nurses | 1.90 | 0.83 | 0.06 | -0.06 | 3.86 |

*The mean difference is significant at the 0.05 level.

Supplementary Table S7: Brief-COPE avoidant (maladaptive) subscale regression analysis for significant demographic and Covid-19 exposure variables

|  | Wald Chi-Square | df | P |
| --- | --- | --- | --- |
| (Intercept) | 406.610 | 1 | .000 |
| Role | 11.930 | 2 | .003 |
| Gender | 12.028 | 3 | .007 |
| Seniority | 59.028 | 1 | .000 |
| Covid-19 infection history | 2.180 | 1 | .140 |
| Work in Covid-19 ward | .005 | 1 | .943 |
| Work in Emergency Department | .200 | 1 | .655 |

Dependent Variable: Brief-COPE Avoidant Subscale

Supplementary Table S8: Post-hoc analysis of Brief-COPE religion subscale, pairwise comparisons by role

|  |  | Mean Difference | Std. Error | *p* | 95% Confidence Interval | |
| --- | --- | --- | --- | --- | --- | --- |
|  |  |  |  |  | Lower Bound | Upper Bound |
| Doctors | Nurses | -0.54^*^ | 0.18 | 0.01 | -0.95 | -0.12 |
|  | Radiographers | 0.00 | 0.33 | 1.00 | -0.77 | 0.77 |
| Nurses | Doctors | 0.54^*^ | 0.18 | 0.01 | 0.12 | 0.95 |
|  | Radiographers | 0.54 | 0.33 | 0.23 | -0.24 | 1.31 |
| Radiographers | Doctors | 0.00 | 0.33 | 1.00 | -0.77 | 0.77 |
|  | Nurses | -0.54 | 0.33 | 0.23 | -1.31 | 0.24 |

*The mean difference is significant at the 0.05 level.

Supplementary Table S9: Post-hoc analyses of significant differences in perceptions scales; pairwise comparisons by roles

|  |  |  |  |  |  | 95% Confidence Interval | |
| --- | --- | --- | --- | --- | --- | --- | --- |
|  |  |  | Mean Difference | Std. Error | *p* | Lower Bound | Upper Bound |
| Health fear | Doctors | Nurses | -0.44^*^ | 0.11 | 0.00 | -0.69 | -0.18 |
|  |  | Radiographers | -0.80^*^ | 0.20 | 0.00 | -1.27 | -0.33 |
|  | Nurses | Doctors | 0.44^*^ | 0.11 | 0.00 | 0.18 | 0.69 |
|  |  | Radiographers | -0.37 | 0.20 | 0.16 | -0.84 | 0.11 |
|  | Radiographers | Doctors | 0.80^*^ | 0.20 | 0.00 | 0.33 | 1.27 |
|  |  | Nurses | 0.37 | 0.20 | 0.16 | -0.11 | 0.84 |
| Job stress | Doctors | Nurses | -0.53^*^ | 0.11 | 0.00 | -0.79 | -0.26 |
|  |  | Radiographers | -0.72^*^ | 0.21 | 0.00 | -1.20 | -0.23 |
|  | Nurses | Doctors | 0.53^*^ | 0.11 | 0.00 | 0.26 | 0.79 |
|  |  | Radiographers | -0.19 | 0.21 | 0.62 | -0.68 | 0.29 |
|  | Radiographers | Doctors | 0.72^*^ | 0.21 | 0.00 | 0.23 | 1.20 |
|  |  | Nurses | 0.19 | 0.21 | 0.62 | -0.29 | 0.68 |
| Social isolation and avoidance | Doctors | Nurses | -0.48^*^ | 0.12 | 0.00 | -0.76 | -0.20 |
|  |  | Radiographers | -0.85^*^ | 0.22 | 0.00 | -1.36 | -0.34 |
|  | Nurses | Doctors | 0.48^*^ | 0.12 | 0.00 | 0.20 | 0.76 |
|  |  | Radiographers | -0.37 | 0.22 | 0.21 | -0.88 | 0.14 |
|  | Radiographers | Doctors | 0.85^*^ | 0.22 | 0.00 | 0.34 | 1.36 |
|  |  | Nurses | 0.37 | 0.22 | 0.21 | -0.14 | 0.88 |

*The mean difference is significant at the 0.05 level.
